# A glance at quality score: implication for *de novo* transcriptome reconstruction of Illumina reads

**DOI:** 10.3389/fgene.2014.00017

**Published:** 2014-02-12

**Authors:** Stanley Kimbung Mbandi, Uljana Hesse, D. Jasper G. Rees, Alan Christoffels

**Affiliations:** ^1^South African Medical Research Council Bioinformatics Unit, South African National Bioinformatics Institute, University of the Western CapeBellville, South Africa; ^2^Biotechnology Platform, Agricultural Research CouncilOnderstepoort, South Africa

**Keywords:** RNA-Seq, quality score, Trinity, transcriptome reconstruction, truncated transfrags, Oases

## Abstract

Downstream analyses of short-reads from next-generation sequencing platforms are often preceded by a pre-processing step that removes uncalled and wrongly called bases. Standard approaches rely on their associated base quality scores to retain the read or a portion of it when the score is above a predefined threshold. It is difficult to differentiate sequencing error from biological variation without a reference using quality scores. The effects of quality score based trimming have not been systematically studied in *de novo* transcriptome assembly. Using RNA-Seq data produced from Illumina, we teased out the effects of quality score based filtering or trimming on *de novo* transcriptome reconstruction. We showed that assemblies produced from reads subjected to different quality score thresholds contain truncated and missing transfrags when compared to those from untrimmed reads. Our data supports the fact that *de novo* assembling of untrimmed data is challenging for de Bruijn graph assemblers. However, our results indicates that comparing the assemblies from untrimmed and trimmed read subsets can suggest appropriate filtering parameters and enable selection of the optimum *de novo* transcriptome assembly in non-model organisms.

## INTRODUCTION

Ultra-high throughput or next generation sequencing (NGS) technologies generates a considerable amount of data. This is desirable for single-nucleotide resolution of the genome and underlying expressed transcriptional units. Their application in sequencing the transcriptome is facilitated by parallel development of reference free assembly algorithms that typically depend on the de Bruijn graph (Martin and Wang, 2011). This has resulted in an increase in the number of published transcriptome assemblies for non-model organisms. However, *de novo* assembly is based on approximate computation, which is impeded by random variations in sampling (bias in reads) and sequencing errors. Sequencing errors introduces false *k*-mers which increases the computational demands for graph resolution and the runtime of assembly algorithms. It is difficult to distinguish between sequencing errors from biological variation without a reference (Garber et al., 2011), since variation becomes dominant with volume of sequence data (Conway and Bromage, 2011). In addition, sampling methods aimed at enriching protein-coding (mRNA) transcripts are overwhelmed by bulk amounts of non-coding RNA (Cui et al., 2010) and immature mRNA with incompletely spliced introns (Garber et al., 2011). For researchers who outsource sequencing services, they do not have access to quality filtering tools embedded in NGS platforms (Cox et al., 2010). We can broadly identify two categories of pre-processing tools that address read usability: error correction and filtering/trimming algorithms which have emerged in response to low quality data. Error correction approaches have been largely applied on genomic reads, e.g, Coral (Salmela and Schröder, 2011) and Quake (Kelley et al., 2010) rely on multiple alignments in *k*-mer space and edit distance respectively to correct reads. Error correction maximizes the quantity of reads for downstream analyses but may reinforce errors and eliminate genuine reads with low frequency *k*-mer (Martin and Wang, 2011). Only recently has error correction been applied to RNA-Seq data where the SEECER algorithm relies on a *k*-mer profile Hidden Markov model (Le et al., 2013). However, MacManes and Eisen (2013) compared error correction tools on RNA-Seq data and showed that Reptile (Yang et al., 2010) performed best with *de novo* transcriptome assembly of error corrected reads. Quality score based-trimming approaches are predominantly used, but they often lead to significant loss of data (Le et al., 2013) and are extremely subjective. Reads are often trimmed in varying modes: ConDeTri (Smeds and Künstner, 2011) trims the reads from the 5′, 3′ or both ends over a defined number of bases (window) or per base and tools such as FASTX-toolkit (http://hannonlab.cshl.edu/fastx_toolkit/) will retain or discard an entire read after assessing quality over a fraction of bases in the read. On the other hand, read content artifacts such as sequencing adaptors and ribosomal RNA may need additional heuristics requirements for pre-processing. The effects of quality score based trimming and artifact removal have not been systematically addressed with respect to the quality of *de novo* assembly derived transcribed fragments. Using NGS data, we compared reference free transcriptome assemblies derived from various categories of quality trimmed reads: with and without artifact removal. Although, the RNA sample used is non-synthetic, we focused on the attributes of the assemblies rather than the biological relevance of the RNA source. We report our findings and propose that caution must be exercised when applying quality filters prior to *de novo* assemblies and that comparing the assemblies of untrimmed and subcategories of trimmed reads could provide an optimal quality score threshold for each read.

## MATERIALS AND METHODS

### DATASETS

Publicly available RNA-Seq data (SRR100067) and the genome assembly (accession AABX00000000) for wild type* Neurospora crassa* 74-OR23-1VA were obtained from the NCBI, http://www.ncbi.nlm.nih.gov/Traces/sra and http://www.ncbi.nlm.nih.gov/Traces/wgsnih.gov/Traces/wgs respectively. Predicted coding sequences (CDS) for *N. crassa* were downloaded from http://fungidb.org release 2.0. In addition, the *Venturia inaequalis* draft genome version 1.0 (Hesse et al., 2013), two lanes of 100 bp paired-end and one lane of 75 bp single-end Illumina data were procured from RNA from a host free culture of *V*. *inaequalis*. The datasets and scripts can be accessed via ftp://ftp.sanbi.ac.za/quality.trimming and https://bitbucket.org/Kimbung/hsp.ratio.

### PRE-PROCESSING RNA-Seq DATA

The raw RNA-Seq data from *N. crassa* was trimmed with a typically used minimum PHRED quality score threshold of 20 (Q20) and 10 (Q10) using ConDeTri, with modification (Smeds and Künstner, 2011) from the 3′-end to represent datasets one and two respectively. For *V. inaequalis*, we generated six categories of quality trimmed or filtered reads as follows: (i) Low quality bases were removed at the 3′-end of each read with a PHRED quality score below 20 or 10 representing datasets one and two respectively, (ii) Potential remnants of adapter sequences were removed using FLEXBAR (Dodt et al., 2012) followed by trimming low quality bases with a PHRED quality score below 20 or 10 that represents datasets three and four, (iii) adapter sequences only removed with FLEXBAR to create dataset five. A minimum read length of 36 bp was used for categories 1–5. A sixth category of pre-processed reads was obtained using the FASTX-toolkit by filtering reads where more than 80% of their bases have a PHRED quality less than 10.

### *DE NOVO* ASSEMBLY

Reference free transcriptome reconstruction with the untrimmed and trimmed *N. crassa* datasets was performed with Trinity (release 2012-06-08; *k*-mer 25; Grabherr et al., 2011). For comparison, Oases (version 0.2.06; Schulz et al., 2012) was used to generate assemblies with various *k*-mers (19–35). *V. inaequalis* datasets were assembled only with Trinity. In all cases, only default assembly parameters were used. Transfrags (TF) ≥ 100 bp were kept for downstream analysis.

### COMPARING ASSEMBLIES

To avoid inflation in alignment or assembly statistics, each assembly was checked for redundant TF using a PERL script to remove exact matches. We aligned the TF from *N. crassa* generated with Q20 (one) and untrimmed reads to the genome with GMAP version 2013-10-04 (Wu and Watanabe, 2005). The following parameters described by Kupfer et al. (2004) were used: min-intron length = 20, max-intron length = 2000, total length = 5904. The total intron length per gene was estimated for *N. crassa* from http://fungi.ensembl.org release-17. The aligned TFs were filtered at high stringency of 95% identity and 95% coverage. TFs from untrimmed reads that did not overlap with those from trimmed reads were verified against predicted CDS loci and recorded as missing annotations using in house PERL scripts for post-processing GMAP alignments. TF derived for the *V. inaequalis* untrimmed and trimmed (category one) reads were aligned to the *V. inaequalis *draft genome using exonerate version 2.2.0 (Slater and Birney, 2005) with the following parameters: model est2genome, maxintron = 5000. Coordinates for best alignment locations were considered and visualized with Gbrowse (http://gmod.org/wiki/GBrowse). The proteins from UniProt Knowledgebase (FUNGI) release 2013_02 (The UniProt Consortium: http://www.uniprot.org) were searched against each customizable databases of TF****assembled from untrimmed and trimmed *V. inaequalis* reads with BLAST+ (Camacho et al., 2009).* N. crassa *TF produced with Trinity from both trimmed and untrimmed reads were searched against UniProt* N. crassa *proteins (*E*-value: 10e^-^^10^). Counts of number of unique high scoring segment pairs (HSP) were computed. The ratio of the length of the HSP to known Uniprot annotated proteins (hereafter referred to as HSP ratio) was generated with a series of in house PERL scripts and UNIX commands for each dataset. HSP ratio represents how well TF were reconstructed. Non-parametric analysis was applied to HSP ratios across read categories and the differences between the read pre-processing approaches was assessed *post hoc* using Agricolae package version 1.1-1 (de Mendiburu, 2012).

## RESULTS

**FIGURE 1:**
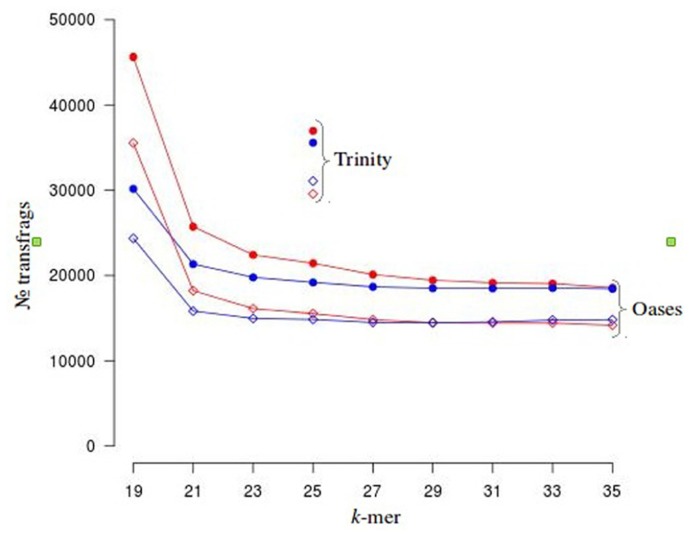
**Distribution of unique (solid circles) and overlapping (diamond shaped) transfrags (TF) from *Neurospora crassa.*** TF from untrimmed and trimmed reads that map to common a genomic loci can be considered as overlapping. Below *k*-23, there is considerable difference in the number of unique and overlapping TF between the trimmed and untrimmed categories. TF from untrimmed and trimmed reads are represented in red and blue, respectively.

**FIGURE 2:**
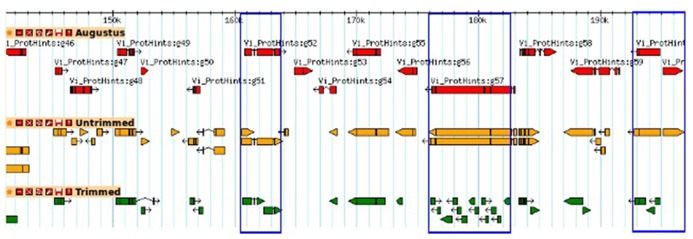
**A GBrowse snapshot of predicted genes and transfrags (TFs) for *V. inaequalis***.* Ab initio* gene predictions are shown in red. TFs produced by Trinity with untrimmed and trimmed (category one) reads are shown in orange and green, respectively.

To investigate the potential side effects of quality based trimming and artifact removal on *de novo* transcriptome assembly, we analyzed datasets from a model (*N. crassa*) and non-model organism (*V. inaequalis*). A summary of read counts for each category of untrimmed and trimmed reads is shown in **Table 1**. More reads are removed when quality based trimming is preceded by adapter removal compared to doing the reverse. The percentage of trimmed reads ranged from 35 to 88%. Out of ~134 Gb *V. inaequalis* untrimmed reads, quality trimming preceded with adapter removal retained the smallest amount of reads. When comparing assemblies from various categories of reads, we note that the number of unique TF from untrimmed reads is always higher than those from trimmed reads irrespective of the assembler and dataset used (**Figure 1**). For *N. crassa* TFs, this is much more profound at lower *k*-mers. A similar trend is observed with the number of TFs, derived from untrimmed and trimmed reads that map to the same genomic loci. TFs produced with untrimmed reads recovered a higher number of known *N. crassa* proteins than those from the trimmed reads (**Table 1**). A total of 521 known gene loci were identified in *N. crassa *that overlapped with TFs derived from untrimmed but not trimmed reads. Transcriptome assembly statistics for each category of quality trimmed reads and the HSP ratios**are shown in **Table 1**. The number of unique TFs is comparable among all assemblies for each organism. Untrimmed reads generated the largest number of TFs and identified the largest numbers of known Uniprot**proteins. Sequence similarity search identified 791 proteins that were present in all *V. inaequalis* assemblies. For *N. crassa*, 6218 proteins were common to all assemblies generated with Trinity. Kruskal–Wallis one-way analysis of variance suggest that quality score base pre-processing had a significant effect on TF quality in both *N. crassa* (*p* = 0.002999) and *V. inaequalis* (*p* < 2.2e-16) data. The mean and median HSP ratios for TF from untrimmed reads were slightly higher than those from trimmed reads for both *N. crassa* and *V. inaequalis*. In addition, the untrimmed datasets has the least variation (**Table 1**). Multiple comparisons testing between HSP ratio is show in **Table 1**. *Post hoc* analysis indicated that the more aggressive Q20 trimming, produced TF of inferior quality compare to the Q10. TF from Q10 and the untrimmed reads yielded not significant different in HSP ratio. Groups with the same letters are not statistically different. Category one and two trimming strategies were significantly different than the other five categories (*p* < 0.01), for *V. inaequalis*. In both *N. crassa* and *V. inaequalis* datasets, TF from untrimmed reads produced higher N50 values. Visual assessment of aligned *V. inaequalis *TF from untrimmed and trimmed reads (category two), reveals missing TF and incomplete TF reconstruction in the latter as shown in **Figure 2**.

**Table 1:** Attributes of transfrags produced with Trinity.

Organism	Read category	No of reads retained	No of unique TF	N50	No of unique HSP	Median HSP ratio	Mean HSP ratio	SD of HSP ratio	Groups at α= 0.01 (*Post hoc*
*N. crassa*	Untrimmed	62,602,096	36964	2557	6773	0.999	0.862	0.212	a
	One	51,630,864	35578	2441	6668	0.992	0.845	0.226	b
	Two	55,155,297	35614	2532	6757	0.997	0.856	0.217	ab
*V. inaequalis*	Untrimmed	134,340,808	45449	1502	923328	0.964	0.859	0.205	a
	One	47,261,404	42325	540	686887	0.879	0.773	0.242	c
	Two	64,617,759	43832	696	760648	0.919	0.805	0.231	b
	Three	67,136,546	38645	979	810854	0.950	0.834	0.225	a
	Four	93,862,916	40311	1237	868775	0.960	0.848	0.214	a
	Five	92,491,510	46166	946	840307	0.949	0.835	0.220	a
	Six	101,402,320	43346	1402	907814	0.964	0.855	0.209	a

## DISCUSSION AND CONCLUSION

In this study, we teased apart the effects of quality based trimming and artifact removal on the quality of *de novo* transcriptome assembly. Quality based trimming approaches are routinely applied on reads generated from NGS platforms. Initial analysis by Garg et al. (2011) suggested that this procedure improved *de novo* transcriptome assembly. However the choice of per base quality score for trimming is subjective and there is no consensus on quality filtering/trimming thresholds since the quality score distribution is non-uniform across samples and the technologies for sequencing are constantly evolving. In addition, the study by Garg et al. (2011) employed a genome assembler which is not suitably optimized for transcriptome reconstruction and this could have had an impact on the interpretation of their results. We observed that, adapter removal was more efficient when performed prior to quality based-trimming. When reads are quality trimmed prior to adapter removal, the sequences may become too short for substring recognition. The higher median and mean HSP ratios and the number of Uniprot identified *V. inaequalis* proteins, suggest that TF derived proteins from assembled untrimmed reads aligned with better quality than those from trimmed reads. Additional support for this observation is revealed by the number of missing annotations in TFs from trimmed *N. crassa* reads. This corroborates anecdotal observation that quality trimming of reads can produce poor assemblies (Paszkiewicz and Studholme, 2010). Untrimmed reads result in more contiguous assemblies, which is probably due to a larger number of paired reads that provide support for connected edges in the de Bruijn graph. Quality trimming affects the quantity of usable reads and for each expression level there is a spectrum of parameters (typically *k*-mer) for optimal transcript assembly (Schulz et al., 2012). In non-model organism, there is an optimal number of reads balancing coverage and errors (Francis et al., 2013) and aggressive trimming or filtering strategies are likely to affect the coverage dynamics. By applying various trimming or filtering approaches, the number of reads appropriate for assembly is achievable when gaged correctly with an suitable metric such as HSP ratio for evaluating the assembly. While quality based trimming is routinely applied prior to *de novo* transcriptome assembly, our analyses suggest that this could lead to missing annotations and incomplete transcript reconstruction. As such, caution must be exercised given that quality score thresholds for read trimming or filtering are subjective. Promiscuous application of quality score based trimming and or filtering should be gaged and additional effective heuristics assessment of transcript reconstruction be applied for each trimming criteria. Furthermore, our analyses demonstrate that HSP ratio in addition to N50 can assist in selecting the optimal transcriptome assembly.

## AUTHOR CONTRIBUTIONS

Stanley Kimbung Mbandi conceived and designed the experiments, performed the experiments, analyzed the data and wrote the manuscript. Uljana Hesse performed transcript visualization. D. Jasper G. Rees provided RNA-Seq data for *V. inaequalis* and critically evaluated the manuscript. Alan Christoffels provided reagents, materials and supervision. Stanley Kimbung Mbandi, Uljana Hesse, D. Jasper G. Rees, and Alan Christoffels approved the final manuscript.

## Conflict of Interest Statement

The authors declare that the research was conducted in the absence of any commercial or financial relationships that could be construed as a potential conflict of interest.
